# A Genetic Variant in Vitamin B12 Metabolic Genes That Reduces the Risk of Congenital Heart Disease in Han Chinese Populations

**DOI:** 10.1371/journal.pone.0088332

**Published:** 2014-02-12

**Authors:** Jue Wang, Jian-Yuan Zhao, Feng Wang, Qian-Qian Peng, Jia Hou, Shu-Na Sun, Yong-Hao Gui, Wen-Yuan Duan, Bin Qiao, Hong-Yan Wang

**Affiliations:** 1 The State Key Laboratory of Genetic Engineering and MOE Key Laboratory of Contemporary Anthropology, School of Life Sciences, Fudan University, Shanghai, China; 2 Children’s Hospital Shanghai, Fudan University, Shanghai, China; 3 CAS-MPG Partner Institute for Computational Biology, Shanghai Institutes for Biological Sciences, Chinese Academy of Sciences, Shanghai, China; 4 Institute of Cardiovascular Disease General Hospital of Jinan Military Region, Jinan, China; 5 The Institutes of Biomedical Sciences, Fudan University, Shanghai, China; Johns Hopkins University, United States of America

## Abstract

**Background:**

Genome-wide association studies on components of the one-carbon metabolic pathway revealed that human vitamin B12 levels could be significantly influenced by variationsinthefucosyltransferase 2 (*FUT2*), cubilin (*CUBN*), and transcobalamin-I (*TCN1*) genes. An altered vitamin B12 level is an important factor that disturbs the homeostasis of the folate metabolism pathway, which in turn can potentially lead to the development of congenital heart disease (CHD). Therefore, we investigated the association between the variants of vitamin B12-related genes and CHD in Han Chinese populations.

**Methods and Results:**

Six variants of the vitamin B12-related genes were selected for analysis in two independent case-control studies, with a total of 868 CHD patients and 931 controls. The variant rs11254363 of the *CUBN* gene was associated with a decreased risk of developing CHD in both the separate and combined case-control studies. Combined samples from the two cohorts had a significant decrease in CHD risk for the G allele (OR = 0.48, *P* = 1.7×10^−5^) and AG+GG genotypes (OR = 0.49, *P* = 4×10^−5^), compared with the wild-type A allele and AA genotype, respectively.

**Conclusions:**

Considering the G allele of variant rs11254363 of the *CUBN* gene was associated with an increased level of circulating vitamin B12. This result suggested that the carriers of the G allele would benefit from the protection offered by the high vitamin B12 concentration during critical heart development stages. This finding shed light on the unexpected role of *CUBN* in CHD development and highlighted the interplay of diet, genetics, and human birth defects.

## Introduction

Congenital heart disease (CHD) is one of the most common congenital birth defects and is among the leading causes of infant death worldwide. CHD takes place during early embryogenesis and requires interactions between genetic and environmental factors. Over the past decades, a series of clinical and basic research studies showed that the folate metabolism pathway is involved in the development of CHD. Peri-conceptual folic acid supplementation could prevent fetal CHD, and folate deficiency in a pregnant woman potentially contributes to CHD in the developing embryo [1], [2]. Vitamin B12, or cobalamin, plays an important role in folate metabolism. In the form of methylcobalamin, this vitaminparticipatesin the folate-dependent methylation of homocysteine to form methionine in the presence of the enzyme methionine synthase. Therefore, vitamin B12 levels and the genes involved in vitamin B12 metabolism may affect the balance of folate metabolismand ultimatelycontribute to the development of CHD through the disruptedfolate metabolism pathway.

There are tworecently published genome-wide association studies (GWAS) and a meta-analysis study of those GWAS scans, that investigate the genetic factors that affect circulating vitamin B12 [3]–[5]. The results of these GWAS showed that the plasma vitamin B12 concentration could be influenced by the variants of the fucosyltransferase 2 (*FUT2*), cubilin (*CUBN*) gene, and transcobalamin-I (*TCN1*) genes [6], [7]. It was determined that the imbalanced folate metabolism pathway might lead to CHD and that the altered plasma vitamin B12 levels could disturb the homeostasis of the folate metabolism pathway [8], [9]. When GWAS studies determined that vitamin B12 levels could be influenced by genetic variants, we considered whether a relationship exists between the identified variants and the risk of CHD. We therefore hypothesized that fetuses with vitamin B12-related *FUT2*, *CUBN*, or *TCN1* variants were vulnerable to the challenge of a maternal vitamin B12 deficiency and were susceptible to the risk of CHD. Hence, we investigated the association between the vitamin B12-related variants and CHD in Han Chinese populations.

## Materials and Methods

### Study Subjects

All of the study protocols were reviewed and approved by the ethics committee of the School of Life Sciences, Fudan University, and written consent forms were obtained from parents and/or patients prior to commencing the study. We analyzed samples in two independent case-control groups that have been described previously [9]–[11]. The Shanghai group consisted of 304 CHD patients and 321 matched controls who were enrolled between August 2008 and February 2011 from the Children’s Hospital of Fudan University (Shanghai, China). The Shandong group consisted of 564 CHD patients and 610 controls recruited between August 2008 and January 2011 at the Cardiovascular Disease Institute, General Hospital of Jinan Military Command (Jinan, Shandong Province, China). All of the controls were non-CHD outpatients from the same geographical area who were matched to the affected individuals in terms of age and sex over the same time period (Table S1). All of the subjects were genetically unrelated ethnic Han Chinese. The CHD patients who manifested additional syndromes or who had a positive family history of CHD in a first-degree relative (parents, siblings, and children) were excluded.

### SNP Selection and Genotyping

Genomic DNA was isolated from venous blood with conventional reagents. The specific regions of the *FUT2*, *CUBN*, and *TCN1* genes were amplified from genomic DNA using a polymerase chain reaction (PCR). The polymorphisms in *FUT2* (rs602662, rs601338 and rs492602), *CUBN* (rs1801222 and rs11254363), and *TCN1* (rs526934) that were significantly associated with vitamin B12 concentration in the GWAS cohorts were selected by genotyping using the SnaPshot Kit (ABI, Foster City, CA, USA). The samples for genotyping were run on an ABI 3730 automated sequencer and analyzed using Peakscan software. For each polymorphism, 32 samples were selected randomly to repeat with SnaPshot genotyping, the concordant rates were 100%. All of the primer pair DNA sequences are listed in Table S2.

### Statistical Analysis

The power evaluation of the sample size was calculated by Quanto program. The differences in the qualitative demographic features and the allelic and genotypic frequencies between the CHD cases and the controls were compared using the χ^2^ test and Fisher’s exact test with SPSS 15.0 software (SPSS, Chicago, IL). The Hardy-Weinberg equilibrium among the controls was also tested using the χ^2^ test. To evaluate the associations between the genotypes and CHD risk, odds ratios (ORs) and 95% confidence intervals (CIs) were calculated using unconditional logistic regression analysis with adjustment for age and sex. The estimation of haplotype frequency and the analysis of the associations between different haplotypes and CHD risk were performed using the SNPstats web tool (http://bioinfo.iconcologia.net/snpstats/start.htm) with adjustments made for age and sex. All of the statistical tests were two-tailed with *P*<0.05 as the significance level.

## Results

We genotyped all 6 polymorphisms in 304 cases versus 321 controls in the Shanghai group and in 564 cases versus 610 controls in the Shandong group. The genotype frequencies of all of the polymorphisms were in accordance with the Hardy-Weinberg equilibrium among the control subjects (*P*>0.05). The allelic and genotypic frequencies of the 6 polymorphisms are listed in Table 1 and Table S3 in the online supplement. The minor allele frequency (MAF) of each polymorphism among our subjects was consistent overall with the published data for the Han Chinese population in the dbSNP database.

**Table 1:** The SNPs investigated in the association study.

SNP ID	Gene	Base change	Location	Group	MAF			Call rate	Genotype*P* ^b^	Genotype*P* ^c^	HWE*P* ^d^
					Control	Case	Database^a^				
rs602662	FUT2	G>A	Exon2	Shanghai	0.005	0.010	0.012	99.5%	0.21	0.44	1.00
				Shandong	0.013	0.020		99.6%	0.23	0.24	1.00
rs601338	FUT2	G>A	Exon2	Shanghai	0.005	0.007	0.012	99.2%	0.46	0.84	1.00
				Shandong	0.011	0.018		99.4%	0.21	0.22	1.00
rs492602	FUT2	A>G	Exon2	Shanghai	0.005	0.005	0.012	98.4%	0.93	1.00	1.00
				Shandong	0.011	0.019		99.7%	0.16	0.15	1.00
rs1801222	CUBN	G>A	Exon8	Shanghai	0.222	0.220	0.122	98.7%	0.32	0.32	0.87
				Shandong	0.150	0.173		99.4%	0.25	0.21	1.00
rs11254363	CUBN	A>G	Intron52	Shanghai	0.035	0.007	0.037	99.4%	**0.0018**	**9.7×10^−4^**	0.31
				Shandong	0.070	0.041		99.2%	**0.0019**	**0.0072**	0.20
rs526934	TCN1	A>G	Intron8	Shanghai	0.228	0.289	0.186	98.2%	**0.0059**	**0.0084**	0.87
				Shandong	0.246	0.258		93.6%	0.73	0.78	0.25

a MAF, minor allele frequency from the HapMap database for the CHB population. The difference in the genotype distributions between the case and control subjects in the co-dominant model was estimated by ^b^ *P*value for the chi-square test and ^c^ *P*value for Fisher’s exact test, respectively. ^d^ *P*value for the Hardy-Weinberg equilibrium test in the control subjects. Additional detailed genotype frequencies are presented in Table S2.

In our association study, we found that the allelic and genotypic distribution of only one SNP (rs11254363, located in the intron 52 of the *CUBN* gene) was significantly different between the CHD patients and the controls in both case/control cohorts (Table 1). The G allele was significantly associated with reduced CHD risk in both Shanghai (OR = 0.19, 95% CI = 0.06–0.54, *P* = 0.0003) and Shandong (OR = 0.57, 95% CI = 0.39–0.82, *P* = 0.002) (Table 2). We investigated the genotypic association between rs11254363 and CHD in 4 genetic models: co-dominant, dominant, recessive and over-dominant. In both cohorts, the most statistically significant results were observed in the dominant model (Shanghai: *P* = 5×10^−4^, Shandong: *P* = 4×10^−4^) (Table 2 and Table S4 in the online supplement). Compared with the wild-type homozygous AA genotype carriers, the individuals carrying the heterozygous AG or homozygous GG genotypes had asignificant protective effect against developing CHD in the Shanghai group (OR = 0.19, 95% CI = 0.06–0.56) and the Shandong group (OR = 0.44, 95% CI = 0.28–0.70) (Table 2). The combined samples by pooling the two groups generated a much more significant *P* value in allele distribution (*P* = 1.7×10^−5^) and genotype distribution (*P* = 4×10^−5^). The similar protective effectswere also revealed inthe G allele (OR = 0.48, 95% CI = 0.34–0.68) and the AG+GG genotypes (OR = 0.49, 95% CI = 0.34–0.69), compared with wild type allele or genotype (Table 2). To avoid potential statistical bias induced by χ^2^ test because some expected counts were less than 5, the Fisher’s exact test was performed to verify the association studies. As shown in Table 1, similar *P* value trends were observed in Fisher’s exact test, compared with theχ^2^ test (Table 1).

**Table 2:** Associations between variant rs11254363 of *CUBN* and CHD in two independent case-control studies.

Group	Genotype/Allele	Control	Case	OR (95% CI)^a^	*P*-value^b^
Shanghai	A/A	297 (93.4%)	299 (98.7%)	1.00	**5×10^−4^**
	A/G-G/G	21 (6.6%)	4 (1.3%)	**0.19 (0.06–0.56)**	
	A	614 (96.5%)	602 (99.3%)	1.00	**0.0003**
	G	22 (3.5%)	4 (0.7%)	0.19 (0.06–0.54)	
Shandong	A/A	529 (86.9%)	512 (92.1%)	1.00	**4×10^−4^**
	A/G-G/G	80 (13.1%)	44 (7.9%)	**0.44 (0.28–0.70)**	
	A	1133 (93.0%)	1067 (96.0%)	1.00	**0.002**
	G	85 (7.0%)	45 (4.0%)	0.57 (0.39–0.82)	
Combined	A/A	826 (89.1%)	811 (94.4%)	1.00	**4×10^−5^**
	A/G-G/G	101 (10.9%)	48 (5.6%)	**0.49 (0.34–0.69)**	
	A	1747 (94.2%)	1669 (97.1%)	1.00	**1.7×10^−5^**
	G	107 (5.8%)	49 (2.9%)	0.48 (0.34–0.68)	

a Adjusted for age and sex; ^b^Genotype and allele frequencies in the case and control participants were compared using the χ^2^ test with 2 degrees of freedom (df) and 1 degree of freedom (df), respectively. The association with genotype was evaluated in the dominant genetic model.

A stratified analysis of rs11254363 was performed according to the CHD classifications we described previously [10], [11]. The most statistically significant result was observed for septation defects (*P* = 0.0014) in the dominant model. In addition, with respect to sub-groups of CHD, we observed that the *CUBN* variant was significantly related to VSD (*P* = 0.0028) (Table S5 in the online supplement).

Another *CUBN* SNP rs1801222 investigated in this study was not significantly related to CHD risk (Table 1). These two *CUBN* SNPs (rs1801222 and rs11254363) were not in high linkage disequilibrium (Table S6 in the online supplement). The haplotype analysis showed the most significant protective effect for the *CUBN* haplotype rs1801222C/rs11254363G, which reduced the risk of developing CHD (OR = 0.49, 95% CI = 0.35–0.70, *P* = 1×10^−4^) (Table S7 in the online supplement). However, we presumed that the *CUBN* haplotype rs1801222C/rs11254363G was being driven by the rs11254363 because the same quantitative protective effect was observed between haplotype analysis and single SNP analysis.

We also identified a significant relationship between SNP rs526934 of the *TCN1* gene and the risk of CHD in the Shanghai group (*P* = 0.0059). However, this result was not repeated in the Shandong group (*P* = 0.73). For the rs602662, rs601338, rs492602 variants in the *FUT2* gene, the genotypic distribution of each variant showed no significant difference between the CHD patients and the control subjects in either cohort (Table 1). Our association study also confirmed that all of the variants in the *FUT2* gene were in high linkage disequilibrium (Table S8 in the online supplement), which is in accordance with a previous study [3].

## Discussion

The accumulated evidencesuggests that vitamin B12 plays an important role in embryo heart development. A pregnant woman with a diet low in vitamin B12 has an increased risk of bearing a child with CHD [12]. Some intervention studies have also indicateda protective effect of vitamin B12 against cardiovascular diseases [13]. In addition, a vitamin B12 deficiency will result in high levels of homocysteine, and maternal hyper-homocysteinemia is a risk factor for CHD [14]–[16]. In our previous study, we identified several variants in homocysteine metabolism genes that were associated with the occurrence of CHD [10], [11], [17]. An altered vitamin B12 level is a key co-factor that disturbs the homeostasis of the folate metabolism pathway, which could potentially lead to the development of CHD because of the unguaranteed folate or a vitamin B12 deficiency.

All 6 SNPs that were significantly related to human vitamin B12 concentration inthe GWAS results were directly evaluated for their potential role in CHD in Han Chinese populations. Why was variant rs11254363 in the *CUBN* gene the only variant that was significantly associated with reduced CHD risk in the current case-control study? The *CUBN* gene encodes the intrinsic factor (IF) - vitamin B12receptor (cubilin). A peripheral membrane protein recognizes complexes of IF and vitamin B12 in the distal ileum, where vitamin B12 is taken up by receptor-mediated endocytosis [18]. The G allele of rs11254363 was associated with reduced CHD risk compared with the A allele. The GWAS results showed that the rs11254363 G allele was significantly related to an increased level of vitamin B12 [5]. As a coenzyme, human vitamin B12 is propitious for the maintenance of methionine synthase (MTR) activity, the accumulation of methyl-donor, and the removal of homocysteine. Therefore, a carrier of rs11254363 G alleles would certainly protect a developing embryo against CHD because of the high level of vitamin B12. However, further functional studies on rs11254363 are required to explain how this variant affects the regulation of the *CUBN* gene or the function of the CUBN protein.

Our study confirmed that there are no associations between any variantsof the *FUT2* gene and CHD, even though there were 3 variants of the *FUT2* gene that significantly influenced the vitamin B12 level in the GWAS. This contradiction might be the result of differences in the genetic structure between Han Chinese and European populations. According to the two GWAS reports, in European populations, the average MAFs of rs602662, rs601338 and rs492602 were 0.44, 0.45 and 0.44, respectively, which were much higher than the values we observed in the Han Chinese population. The MAFs of those variants in our study were under 0.02 overall, which were consistent with the published data for the Han Chinese population in the dbSNP database. The low MAFs increased the requirement of sample size. In the power evaluation of the sample size, we observed that all the *FUT2* variants had lower statistical power (Table S9). Expanded sample size should be surveyed to validate this *FUT2* variants association results.

Notably, another GWAS reported that the positive SNPs of the *FUT2* gene in Europeans were not significantly associated with the vitamin B12 level in the Chinese population. Interestingly, other SNPs in the *FUT2* and *FUT6* genes were significantly associated with vitamin B12 levels specifically in the Chinese population [19]. Both the GWAS in the European population and the GWAS in the Chinese population consistently showed that the *FUT2* variants influence the vitamin B12 level. It would be an attractive research aim to explore the *FUT2* variants associated with the risk of CHD, even though the biological function of *FUT2* is currently unknown and there is no information about its role invitamin B12 metabolism. Therefore, it would be valuable to identify new high-frequency genetic markers in the *FUT2* gene in the Han Chinese population and to investigate their relationship with vitamin B12 metabolism and CHD in future studies.

In summary, this initial study focused on evaluating the genetic variants of vitamin B12 metabolism-related genes and found that variant rs11254364 in the *CUBN* gene was significantly associated with the risk of CHD in Chinese populations. Our results accentuated the importance of the vitamin B12 metabolism balance in embryo heart development, provided new insight for risk assessments of common birth defects and shed light on the interplay between nutrients, genetics, and congenital heart disease.

## Supporting Information

Table S1 **Demographic characteristics in CHD cases and controls.**
(DOCX)

Table S2 **DNA sequence of all used primers.**
(DOCX)

Table S3 **The genotype frequency of the selected variants in CHD patients and controls.**
(DOCX)

Table S4 **Association between variant rs11254363 and CHD in different genetic models.**
(DOCX)

Table S5 **Stratification analysis of rs11254363 genotypes according to CHD classification and phenotype.**
(DOCX)

Table S6 **The linkage disequilibrium structure of** ***CUBN*** **gene variants.**
(DOCX)

Table S7 ***CUBN*** **haplotype analysis between CHDs and controls.**
(DOCX)

Table S8 **The linkage disequilibrium structure of** ***FUT2*** **gene variants.**
(DOCX)

Table S9 **The power evaluation of the sample size was calculated by Quanto program.**
(DOCX)
