# Supplementary material for: A Genetic Variant in Vitamin B12 Metabolic Genes That Reduces the Risk of Congenital Heart Disease in Han Chinese Populations
Source: PLoS One. 2014 Feb 12;9(2):e88332. doi: 10.1371/journal.pone.0088332 (PMC3922769; doi:10.1371/journal.pone.0088332)
Supplement: Table S1 — Demographic characteristics in CHD cases and controls. (DOCX) [file pone.0088332.s001.docx]

**Table S1.** Demographic characteristics in CHD cases and controls

| **Variable** | **Cases** | | **Controls** | | ***P* value^a^** |
| --- | --- | --- | --- | --- | --- |
|  | **No.** | **%** | **No.** | **%** |  |
| Stage 1, Shanghai Group | *N*=304 |  | *N*=321 |  |  |
| Age, years (mean±SE) | 5.08±0.31 |  | 4.91±0.22 |  | **0.66** |
| Gender |  |  |  |  | **0.37** |
| Male | 185 | 60.9 | 184 | 57.3 |  |
| Female | 119 | 39.1 | 137 | 42.7 |  |
| Stage 2, Shandong Group | *N*=564 |  | *N*=610 |  |  |
| Age, years (mean±SE) | 6.38±0.26 |  | 6.81±0.14 |  | **0.15** |
| Gender |  |  |  |  | **0.93** |
| Male | 287 | 50.9 | 312 | 51.1 |  |
| Female | 277 | 49.1 | 298 | 48.9 |  |
| Combined samples | *N*=868 |  | *N*=931 |  |  |
| Age, years (mean±SE) | 5.93±0.20 |  | 6.15±0.13 |  | **0.33** |
| Gender |  |  |  |  | **0.64** |
| Male | 472 | 54.3 | 496 | 53.2 |  |
| Female | 396 | 45.7 | 435 | 46.8 |  |
| CHD classification |  |  |  |  |  |
| Conotruncal defects | 121 | 13.9 |  |  |  |
| Septation defects | 747 | 86.1 |  |  |  |
| Isolated CHD phenotype |  |  |  |  |  |
| VSD | 568 | 65.4 |  |  |  |
| ASD | 126 | 14.5 |  |  |  |
| TOF | 98 | 11.3 |  |  |  |

^a^The comparison of age was performed by student T test, and the comparison of gender was performed by 2-tailed χ^2^ test. Date shown in the row of age is means±SE.
