# Supplementary material for: A Genetic Variant in Vitamin B12 Metabolic Genes That Reduces the Risk of Congenital Heart Disease in Han Chinese Populations
Source: PLoS One. 2014 Feb 12;9(2):e88332. doi: 10.1371/journal.pone.0088332 (PMC3922769; doi:10.1371/journal.pone.0088332)
Supplement: Table S2 — DNA sequence of all used primers. (DOCX) [file pone.0088332.s002.docx]

**Table S2.**DNA sequence of all used primers

| PRIMER NAME | SEQUENCE (5’-3’) | PURPOSE |
| --- | --- | --- |
| FUT2-F | CCTCAACATCAAAGGCACTGG | PCR |
| FUT2-R | GTCTCCGCCCGTGAGGTAT | PCR |
| CUBN-F1 | TTTTGACGTGGAATTGTGATAGC | PCR |
| CUBN-R1 | GATTGGCCTTATGGAAATGAAC | PCR |
| CUBN-F2 | CTCTGTGCTTGTGAGCCATC | PCR |
| CUBN-R2 | ACACACAGAGGTTGGCAGAAG | PCR |
| TCN1-F | AGAGTTGAAGGAAGGTGGACAT | PCR |
| TCN1-R | GTGTATTCACTTGCCTAACTGCTC | PCR |
| rs492602 Typing | GGGATGTGGACGATCAATGC | SNaPshot |
| rs601338 Typing | CGGAGGTGGTGGTAGAAGGTC | SNaPshot |
| rs602662 Typing | GGAGAACATTGACACCTCCCAC | SNaPshot |
| rs1801222 Typing | CTGTGATGCTGGGTGGATGT | SNaPshot |
| rs11254363 Typing | GATATTAATTTCTGTATGTGAAATTGTAA | SNaPshot |
| rs526934 Typing | AAATCATGCATTGAATTTCAGG | SNaPshot |
