# Supplementary material for: A Genetic Variant in Vitamin B12 Metabolic Genes That Reduces the Risk of Congenital Heart Disease in Han Chinese Populations
Source: PLoS One. 2014 Feb 12;9(2):e88332. doi: 10.1371/journal.pone.0088332 (PMC3922769; doi:10.1371/journal.pone.0088332)
Supplement: Table S3 — The genotype frequency of the selected variants in CHD patients and controls. (DOCX) [file pone.0088332.s003.docx]

**Table S3.** The genotype frequency of the selected variants in CHD patients and controls

| SNP | Group | Genotype | Control | Case |
| --- | --- | --- | --- | --- |
| rs602662 | Shanghai | G/G | 315 (99.1%) | 300 (98.7%) |
|  |  | G/A | 3 (0.9%) | 2 (0.7%) |
|  |  | A/A | 0 (0%) | 2 (0.7%) |
|  |  | G/G | 594 (97.4%) | 537 (96.1%) |
|  | Shandong | A/G | 16 (2.6%) | 22 (3.9%) |
|  |  | A/A | 0 (0%) | 0 (0%) |
| rs601338 | Shanghai | G/G | 314 (99%) | 300 (99%) |
|  |  | G/A | 3 (1%) | 2 (0.7%) |
|  |  | A/A | 0 (0%) | 1 (0.3%) |
|  | Shandong | G/G | 596 (97.7%) | 537 (96.4%) |
|  |  | G/A | 14 (2.3%) | 20 (3.6%) |
|  |  | A/A | 0 (0%) | 0 (0%) |
| rs492602 | Shanghai | A/A | 315 (99.1%) | 294 (99%) |
|  |  | A/G | 3 (0.9%) | 3 (1%) |
|  |  | G/G | 0 (0%) | 0 (0%) |
|  | Shandong | A/A | 596 (97.7%) | 540 (96.3%) |
|  |  | A/G | 14 (2.3%) | 21 (3.7%) |
|  |  | G/G | 0 (0%) | 0 (0%) |
| rs1801222 | Shanghai | G/G | 195 (60.8%) | 187 (63.2%) |
|  |  | G/A | 110 (34.3%) | 88 (29.7%) |
|  |  | A/A | 16 (5%) | 21 (7.1%) |
|  | Shandong | G/G | 438 (72%) | 387 (69.2%) |
|  |  | G/A | 157 (25.8%) | 151 (27%) |
|  |  | A/A | 13 (2.1%) | 21 (3.8%) |
| rs11254363 | Shanghai | A/A | 297 (93.4%) | 299 (98.7%) |
|  |  | A/G | 20 (6.3%) | 4 (1.3%) |
|  |  | G/G | 1 (0.3%) | 0 (0%) |
|  | Shandong | A/A | 529 (86.9%) | 512 (92.1%) |
|  |  | A/G | 75 (12.3%) | 43 (7.7%) |
|  |  | G/G | 5 (0.8%) | 1 (0.2%) |
| rs526934 | Shanghai | A/A | 192 (59.8%) | 160 (54.6%) |
|  |  | A/G | 112 (34.9%) | 97 (33.1%) |
|  |  | G/G | 17 (5.3%) | 36 (12.3%) |
|  | Shandong | A/A | 313 (57.8%) | 310 (55.7%) |
|  |  | A/G | 191 (35.2%) | 207 (37.2%) |
|  |  | G/G | 38 (7%) | 40 (7.2%) |
