# Supplementary material for: A Genetic Variant in Vitamin B12 Metabolic Genes That Reduces the Risk of Congenital Heart Disease in Han Chinese Populations
Source: PLoS One. 2014 Feb 12;9(2):e88332. doi: 10.1371/journal.pone.0088332 (PMC3922769; doi:10.1371/journal.pone.0088332)
Supplement: Table S4 — Association between variant rs11254363 and CHD in different genetic models. (DOCX) [file pone.0088332.s004.docx]

**Table S4.** Association between variant rs11254363 and CHD in different genetic models

|  | **Model** | **Genotype** | **Control** | **Case** | **OR (95% CI)^a^** | ***P*-value^b^** |
| --- | --- | --- | --- | --- | --- | --- |
| Shanghai | Codominant | A/A | 297 (93.4%) | 299 (98.7%) | 1.00 | **0.0018** |
|  |  | A/G | 20 (6.3%) | 4 (1.3%) | **0.20 (0.07-0.59)** |  |
|  |  | G/G | 1 (0.3%) | 0 (0%) | 0.00 (0.00-NA) |  |
|  | Dominant | A/A | 297 (93.4%) | 299 (98.7%) | 1.00 | **5×10^-4^** |
|  |  | A/G-G/G | 21 (6.6%) | 4 (1.3%) | **0.19 (0.06-0.56)** |  |
|  | Recessive | A/A-A/G | 317 (99.7%) | 303 (100%) | 1.00 | 0.24 |
|  |  | G/G | 1 (0.3%) | 0 (0%) | 0.00 (0.00-NA) |  |
|  | Overdominant | A/A-G/G | 298 (93.7%) | 299 (98.7%) | 1.00 | **8×10^-4^** |
|  |  | A/G | 20 (6.3%) | 4 (1.3%) | **0.20 (0.07-0.59)** |  |
| Shandong | Codominant | A/A | 529 (86.9%) | 512 (92.1%) | 1.00 | **0.0019** |
|  |  | A/G | 75 (12.3%) | 43 (7.7%) | **0.45 (0.28-0.72)** |  |
|  |  | G/G | 5 (0.8%) | 1 (0.2%) | 0.35 (0.04-3.38) |  |
|  | Dominant | A/A | 529 (86.9%) | 512 (92.1%) | 1.00 | **4×10^-4^** |
|  |  | A/G-G/G | 80 (13.1%) | 44 (7.9%) | **0.44 (0.28-0.70)** |  |
|  | Recessive | A/A-A/G | 604 (99.2%) | 555 (99.8%) | 1.00 | 0.36 |
|  |  | G/G | 5 (0.8%) | 1 (0.2%) | 0.38 (0.04-3.64) |  |
|  | Overdominant | A/A-G/G | 534 (87.7%) | 513 (92.3%) | 1.00 | **7×10^-4^** |
|  |  | A/G | 75 (12.3%) | 43 (7.7%) | **0.45 (0.28-0.72)** |  |
| Combined | Codominant | A/A | 826 (89.1%) | 811 (94.4%) | 1.00 | **1×10^-4^** |
|  |  | A/G | 95 (10.2%) | 47 (5.5%) | **0.51 (0.35-0.73)** |  |
|  |  | G/G | 6 (0.6%) | 1 (0.1%) | 0.17 (0.02-1.42) |  |
|  | Dominant | A/A | 826 (89.1%) | 811 (94.4%) | 1.00 | **4×10^-5^** |
|  |  | A/G-G/G | 101 (10.9%) | 48 (5.6%) | **0.49 (0.34-0.69)** |  |
|  | Recessive | A/A-A/G | 921 (99.3%) | 858 (99.9%) | 1.00 | 0.059 |
|  |  | G/G | 6 (0.6%) | 1 (0.1%) | 0.18 (0.02-1.50) |  |
|  | Overdominant | A/A-G/G | 832 (89.8%) | 812 (94.5%) | 1.00 | **2×10^-4^** |
|  |  | A/G | 95 (10.2%) | 47 (5.5%) | **0.51 (0.35-0.73)** |  |

^a^ Adjusted for age, sex; ^b^ Genotype frequencies in case and control participants were compared using χ^2^ test with 2 degrees of freedom (df).
