# Supplementary material for: A Genetic Variant in Vitamin B12 Metabolic Genes That Reduces the Risk of Congenital Heart Disease in Han Chinese Populations
Source: PLoS One. 2014 Feb 12;9(2):e88332. doi: 10.1371/journal.pone.0088332 (PMC3922769; doi:10.1371/journal.pone.0088332)
Supplement: Table S5 — Stratification analysis of rs11254363 genotypes according to CHD classification and phenotype. (DOCX) [file pone.0088332.s005.docx]

**Table S5.** Stratification analysis of rs11254363 genotypes according to CHD classification and phenotype

| **Variable** | **Case number** | ***P* value^a^** | **Association OR (95% CI)^b^** |
| --- | --- | --- | --- |
| **CHD Classification** |  |  | AG-GG vs AA |
| Conotruncal defects | 121 | **0.021** | **0.34 (0.12-0.97)** |
| Septation defects | 747 | **0.0014** | **0.52 (0.34-0.78)** |
| **Detailed phenotype** |  |  |  |
| ASD | 126 | 0.11 | 0.51 (0.21-1.23) |
| VSD | 568 | **0.0028** | **0.51 (0.32-0.81)** |
| TOF | 98 | 0.081 | 0.43 (0.15-1.23) |

ASD, atrial septal defect; VSD, ventricular septal defect; and TOF, tetralogy of Fallot. The controls used were the total 931 combined controls.

^a^P value in dominant genetic model; ^b^ Adjusted for age and sex.
