# Supplementary material for: A Genetic Variant in Vitamin B12 Metabolic Genes That Reduces the Risk of Congenital Heart Disease in Han Chinese Populations
Source: PLoS One. 2014 Feb 12;9(2):e88332. doi: 10.1371/journal.pone.0088332 (PMC3922769; doi:10.1371/journal.pone.0088332)
Supplement: Table S6 — The linkage disequilibrium structure of CUBN gene variants. (DOCX) [file pone.0088332.s006.docx]

**Table S6.** The linkage disequilibrium structure of *CUBN* gene variants

|  | **D' statistic** | | |  |  |  | **R^2^ statistic** | | |  |
| --- | --- | --- | --- | --- | --- | --- | --- | --- | --- | --- |
|  | | rs1801222 | rs11254363 | |  |  | | rs1801222 | rs11254363 | |
| rs1801222 | | . | 0.994 | |  | rs1801222 | | . | 0.01 | |
| rs11254363 | | . | . | |  | rs11254363 | | . | . | |
