# Supplementary material for: A Genetic Variant in Vitamin B12 Metabolic Genes That Reduces the Risk of Congenital Heart Disease in Han Chinese Populations
Source: PLoS One. 2014 Feb 12;9(2):e88332. doi: 10.1371/journal.pone.0088332 (PMC3922769; doi:10.1371/journal.pone.0088332)
Supplement: Table S7 — CUBN haplotype analysis between CHDs and controls. (DOCX) [file pone.0088332.s007.docx]

**Table S7**. *CUBN* haplotype analysis between CHDs and controls

| **No.** | **rs1801222** | **rs11254363** | **Freq (Control)** | **Freq (Case)** | **Freq (Total)** | **OR (95% CI)^a^** | ***P*-value^b^** |
| --- | --- | --- | --- | --- | --- | --- | --- |
| 1 | C | A | 0.7725 | 0.7825 | 0.7753 | 1.00 | --- |
| 2 | T | A | 0.1697 | 0.1891 | 0.181 | 1.08 (0.97-1.27) | 0.39 |
| 3 | C | G | 0.054 | 0.0284 | 0.0438 | 0.49 (0.35-0.70) | 1×10^-4^ |
| 4 | T | G | 0.0038 | 0 | 0 | --- | --- |

^a^Adjusted by age and gender;

^b^*P* value for difference in haplotypes distributions between control and case subjects.
