# Supplementary material for: A Genetic Variant in Vitamin B12 Metabolic Genes That Reduces the Risk of Congenital Heart Disease in Han Chinese Populations
Source: PLoS One. 2014 Feb 12;9(2):e88332. doi: 10.1371/journal.pone.0088332 (PMC3922769; doi:10.1371/journal.pone.0088332)
Supplement: Table S8 — The linkage disequilibrium structure of FUT2 gene variants. (DOCX) [file pone.0088332.s008.docx]

**Table S8.** The linkage disequilibrium structure of *FUT2* gene variants

|  | | **D' statistic** | |  | |  |  | | **R^2^ statistic** | |  | |
| --- | --- | --- | --- | --- | --- | --- | --- | --- | --- | --- | --- | --- |
|  | rs602662 | | rs601338 | | rs492602 |  |  | rs602662 | | rs601338 | | rs492602 |
| rs602662 | . | | 0.9954 | | 0.9954 |  | rs602662 | . | | 0.8647 | | 0.8653 |
| rs601338 | . | | . | | 0.9867 |  | rs601338 | . | | . | | 0.9730 |
| rs492602 | . | | . | | . |  | rs492602 | . | | . | | . |
