# Supplementary material for: A Genetic Variant in Vitamin B12 Metabolic Genes That Reduces the Risk of Congenital Heart Disease in Han Chinese Populations
Source: PLoS One. 2014 Feb 12;9(2):e88332. doi: 10.1371/journal.pone.0088332 (PMC3922769; doi:10.1371/journal.pone.0088332)
Supplement: Table S9 — The power evaluation of the sample size was calculated by Quanto program. (DOCX) [file pone.0088332.s009.docx]

**Table S9.** The power evaluation of the sample size was calculated by Quanto program

| SNP ID | Shanghai | Shandong |
| --- | --- | --- |
| rs602662 | 0.12 | 0.50 |
| rs601338 | 0.05 | 0.56 |
| rs492602 | 0.05 | 0.64 |
| rs1801222 | 0.14 | 0.99 |
| rs11254363 | 0.97 | 0.99 |
| rs526934 | 0.46 | 0.05 |
